# *In vitro* and *in vivo* antitumor potential of carvacrol nanoemulsion against human lung adenocarcinoma A549 cells via mitochondrial mediated apoptosis

**DOI:** 10.1038/s41598-017-18644-9

**Published:** 2018-01-09

**Authors:** Imran Khan, Ashutosh Bahuguna, Pradeep Kumar, Vivek K. Bajpai, Sun Chul Kang

**Affiliations:** 1 Department of Biotechnology, Daegu University, Gyeongsan, Gyeongbuk, 712-714 Republic of Korea; 2 Department of Forestry, North Eastern Regional Institute of Science and Technology, Nirjuli, India; 3 Department of Energy and Materials Engineering, Dongguk University-Seoul, Seoul, Republic of Korea

## Abstract

Carvacrol is present abundantly in the essential oils of many medicinal plants and well known for its numerous biological activities. Since partial solubility in water and physicochemical instability limits its industrial uses, the present study was performed to prepare a carvacrol nanoemulsion (CANE) using an ultrasonication technique and further evaluation of its anticancer potential against human lung adenocarcinoma A549 cells. The nanoemulsion formulation was optimized by varying carvacrol and polysorbate 80 ratios and characterized by dynamic light scattering (DLS), which revealed a negative surface charge with a mean droplet size between 105.5 ± 3.4 to 169.8 ± 4.9 nm. The CANE induced reactive oxygen species (ROS) production in A549 cells, leading to activation of key regulators of apoptosis such as p-JNK, Bax and Bcl2 as well as release of cytochrome C, and activation of the caspase cascade. Suppression of mitochondrial ROS using Mito-TEMPO reversed the apoptotic potential of CANE signifying involvement of mitochondrial ROS in cell death. Beside, CANE displayed a strong antitumor potential *in vivo* using an athymic nude mice model. The results strongly support that CANE induced apoptosis in A549 cells by induction of ROS and could be a promising candidate for lung cancer therapy.

## Introduction

Lung cancer is considered a major global health problem due to increased tobacco smoking and air pollution. A total of 1.8 million cases of lung cancer were reported worldwide in the year 2012 with 1.6 million deaths^1^. Lung cancer is the most common cause of deaths in males and the second most frequent cause of death in females after breast cancer^2^. The survival rate is only 5 years in approximately 85% of the adenocarcinoma patients after diagnosis^3^. Treatment for lung cancer includes surgery, chemotherapy, radiotherapy and palliative care, all of which highly depend upon disease state and patient performance status. However, chemotherapy with a single drug or in combination is the most common therapy to treat lung cancer^4^. Despite much advancement, chemotherapy still proves insufficient to cure cancer, and the side effect exerted by these drugs on the patient^5,6^ and hazards to the environment^7^ limits their use.

Phytochemicals are generally non-toxic in nature, prove effective against many diseases, and provide a safe and effective alternative against cancer^8^. Among phytochemicals, carvacrol, a monoterpenoid phenol, is found abundantly in essential oil of oregano and thyme^9^ and is known to exert many biological effects, including antimicrobial, insecticidal, anti-angiogenic, and anti-tumor activity^10,11^. Of note, the Food and Drug Administration (FDA) has approved the use of carvacrol as a food additive which attests its non-toxic nature^12^. Also, the literature has documented that many natural compounds exert anticancer activity by induction of apoptosis, a principle mechanism of cell death^13^.

Moreover, essential oils and their components are well known for anticancer potential^14^ predominantly by the induction of reactive oxygen species (ROS). ROS are the byproducts of normal cellular metabolism and can be beneficial or harmful depending on the intensity and site of accumulation. Cytosol, endoplasmic reticulum (ER) and mitochondria are the important sources of cellular ROS in most mammalian cells. Abnormally high ROS levels create ER stress with the involvement of three major signaling proteins IRE1-α, PERK and ATF-6. IRE1-α signaling protein is known to phosphorylate JNK which in turn regulates mitochondrial markers such as Bax, Bcl2, and Cyt C leading to caspase-mediated cell death^15^.

In recent years, nanoemulsions (NEs) have gained huge attention due to their wide applicability in pharmaceuticals and other industries^16^. Nano-sized emulsions provide numerous advantages that impose their high absorption due to increased surface area and thus the obvious effects on bioavailability and can be used as a novel drug delivery system and substitute to liposome and vesicle^17^. In addition, NEs protect active components against physicochemical stress and prolong persistence as compared to free drugs, facilitating additional routes such as oral, tropical, and intravenous drug delivery^16,18,19^. Moreover, the solubility of lipophilic compounds can be improved in water in the form of an emulsion which consecutively augment their bioavailability and pharmacokinetic properties^20^.

The present study was designed to formulate a carvacrol nanoemulsion (CANE) using energy generated by ultrasonication and evaluates its mechanism of anticancer action using human lung adenocarcinoma A549 cell line and *in vivo* xenograft mice model.

## Results

### Formulation and characterization nanoemulsion

Mean droplet size and polydispersity index (PDI) of the formulated nanoemulsions were analyzed by dynamic light scattering (DLS), and results are depicted in Table 1. Average droplet size of the three different formulations of CANE considerably decreased with increasing concentration of surfactant (Table 1). PDI determined by DLS of all three combinations of CANE was in the range of 0.134–0.159, which was close to the homogeneity of the preparation (Table 1).

**Table 1:** Physical and chemical properties of formulated CANE after ultrasonication.

Carvacrol:surfactant (v/v)	Average droplet size (nm)	Polydispersity index (PDI)	Zeta potential (mV)
1:1	169.8 ± 4.9	0.141 ± 0.04	−15.5 ± 1.3
1:2	136.8 ± 5.1	0.159 ± 0.05	−17.0 ± 2.0
1:3	105.5 ± 3.4	0.134 ± 0.04	−19.0 ± 1.6

Values with different superscripts in the column differ significantly from each other (*p* < 0.05).

**Figure 1:**
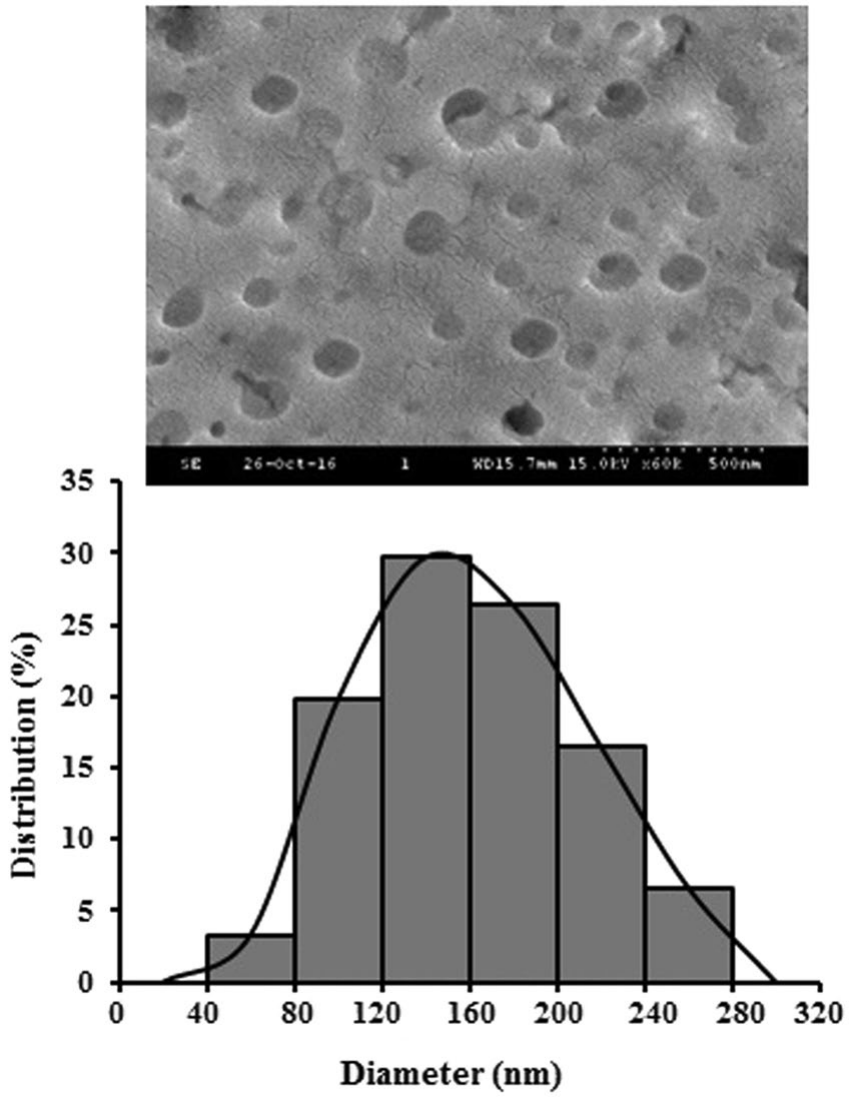
Scanning electron microscopic (SEM) image of CANE (1:3 v/v) and its histogram size distribution [Scale bar = 500 nm].

A higher negative zeta potential (−19 mV) was observed in the NE formed by mixing 1:3 (v/v) carvacrol-polysorbate 80 components as compared to the other two formulations (Table 1). Further, SEM examination revealed a spherical shape with 155 nm average droplet size of CANE (1:3 v/v) (Fig. 1). In all the three formulations, 1:3 (v/v) CANE displayed least size with a high negative zeta potential and thus selected in subsequent assays for evaluation of anticancer studies.

### Cytotoxicity of CANE against A549 cells

**Figure 2:**
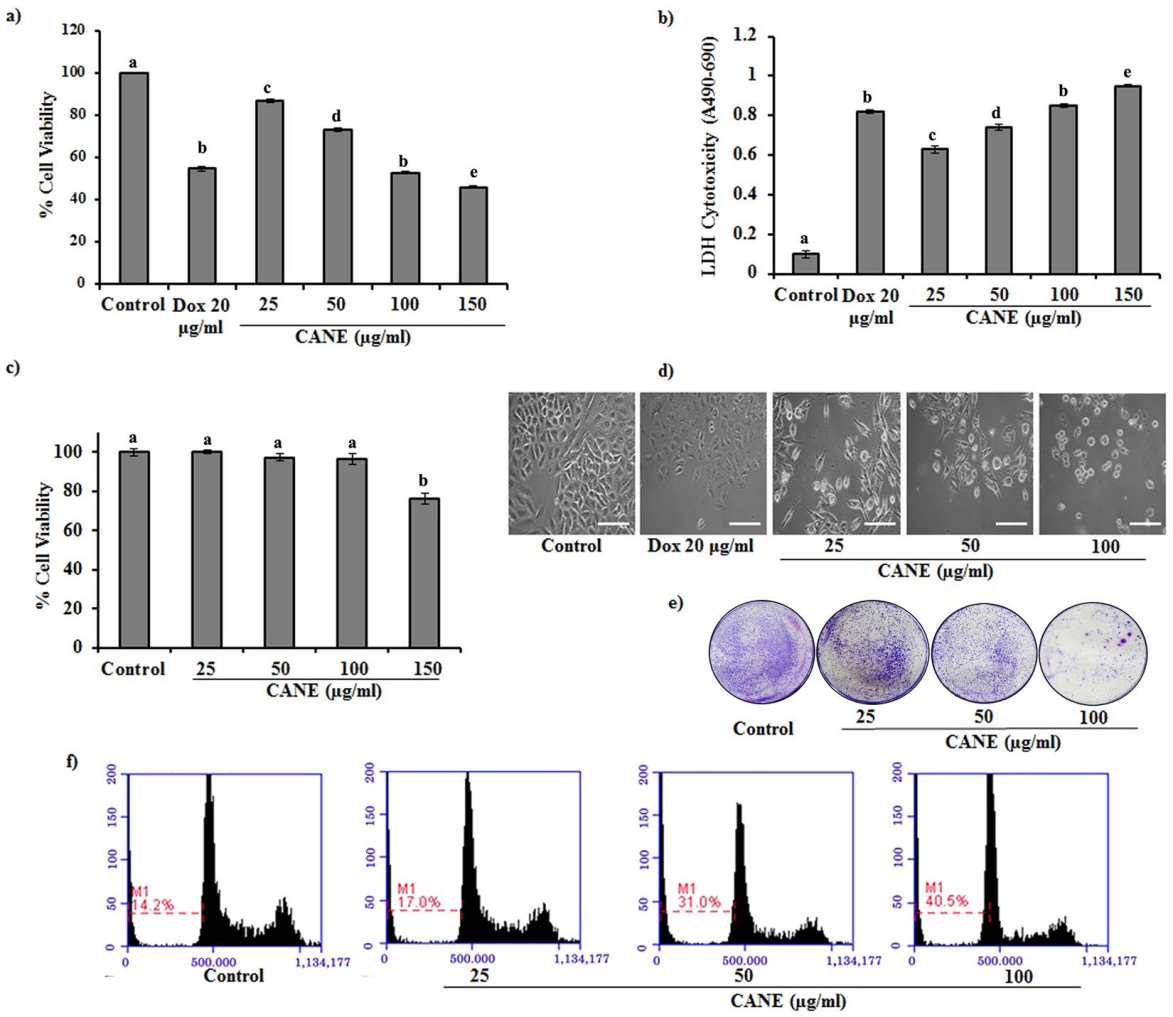
Cytotoxic potential of CANE against A549 cells. (**a**,**b**) MTT and LDH assay, respectively, of CANE (25–150 μg/ml)-treated A549 cells after 24 h of incubation. Docetaxel (20 μg/ml) was used as a positive control while the surfactant-polysorbate 80 mix devoid of carvacrol was also used as a control. (**c**) MTT assay of CANE (25–150 μg/ml)-treated normal bronchial epithelium cells (BEAS-2B). (**d**) Morphological changes in A549 cells after treatment with CANE (25–100 μg/ml). Images were captured at 20X magnification [scale bar = 0.1 mm]. (**e**) Clonogenic assay: A549 cells were cultured in the presence and absence of CANE over 7 days, followed by crystal violet staining. (**f**) Apoptosis was analyzed as the sub-G1 fraction by FACS. Each value in the bar graph represents the mean ± SD of three independent experiments. Values with different superscripts differ significantly from each other (*p* < 0.05).

As depicted in Fig. 2 CANE exhibited a severe cytotoxicity in a dose-dependent manner. MTT assay revealed 52.7% cell viability in CANE (100 µg/ml)-treated cells as compared to untreated cells (Fig. 2a). On the other hand, higher LDH activity, which is a well-established biomarker released by damaged cells, was observed in CANE-treated cells (Fig. 2b). Furthermore, CANE displayed no cytotoxicity up to 100 µg/ml against normal bronchial epithelium cells (BEAS-2B) (Fig. 2c).

As depicted in Fig. 2d, CANE caused loss of cell numbers along with abnormal morphology in A549 cells in comparison to the control cells without treatment. Moreover, a severe effect on colony formation was observed in A549 cells treated with CANE (Fig. 2e).

Cytotoxicity of CANE was finally assessed by quantifying sub-G1 population as confirmed by FACS analysis. As depicted in Fig. 2f, CANE dose-dependently induced the accumulation of sub-G1 population, a well-known apoptotic marker in A549 cells. A maximum of 40.5% sub-G1 cell accumulation was observed at 100 µg/ml of CANE treatment.

### Induction of apoptosis and ROS production in the presence of CANE

**Figure 3:**
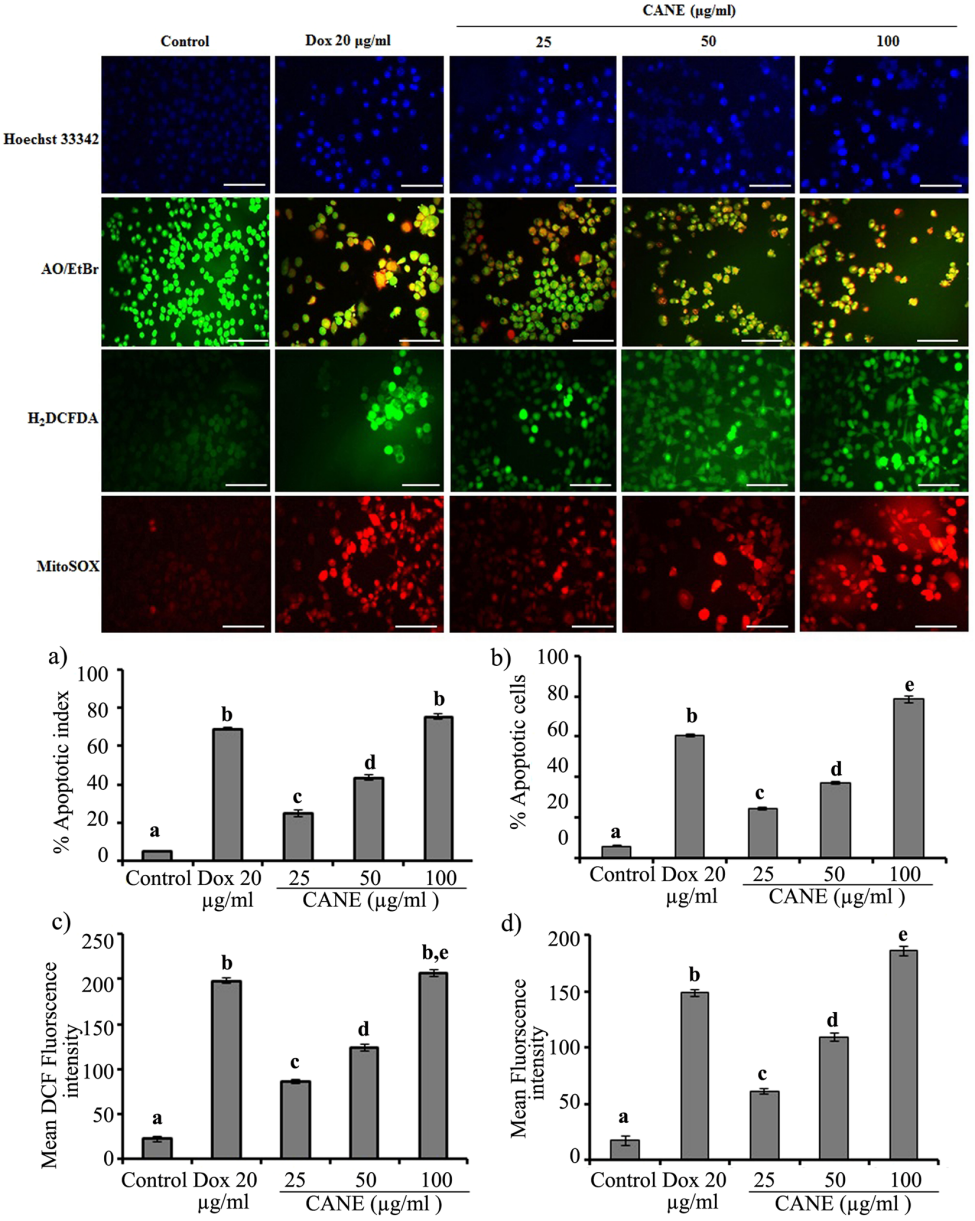
CANE (25–100 μg/ml) induces apoptotic morphology and ROS production in A549 cells after 24 h of incubation. Apoptotic morphology was evaluated by Hoechest and AO/EtBr dual staining. Cytosolic ROS determined by H_2_DCFDA while the Mitochondrial superoxide determined by MitoSOX. Docetaxel (20 μg/ml) was used as a positive control while the surfactant-polysorbate 80 mix devoid of carvacrol was also used as a control. Images were taken at 20X magnification [scale bar = 0.1 mm]. (**a**) Apoptotic index was calculated as the percentage of apoptotic nuclei compared to the total number of cells and is presented as the mean ± SD (n = 10). (**b**) Apoptotic cells determined by AO/EtBr dual staining. (**c**,**d**) Fluorescence intensity of H_2_DCFDA (green color) and MitoSOX (red color) was determined by Image J software. Each value in the graph represents the mean ± SD of three independent experiments. Values with different superscripts differ significantly from each other (*p* < 0.05).

Apoptotic potential of CANE was evaluated by Hoechst staining, which revealed a dose-dependent high percentage of apoptotic nuclei in cells treated with CANE as compared to the control, suggesting its potential role on DNA damage and chromatin condensation (Fig. 3).

Apoptotic cell formation and the stages of apoptosis such as early and late were further confirmed by AO/EtBr dual staining where normal cells uptake AO and appear green while the EtBr enter only through the damaged cells thus appear red. We observed a dose-dependent increase in the apoptotic cells after treatment with CANE as compared to the control (Fig. 3).

Results obtained from H_2_DCFDA staining suggest a dose-dependent elevated intracellular ROS level in CANE treated cells (Fig. 3). In addition, results achieved from the mitochondrial-specific fluorogenic MitoSOX red dye indicated a dose-dependent increase in superoxide production inside mitochondria (Fig. 3).

### Upregulation of apoptotic proteins after CANE treatment

**Figure 4:**
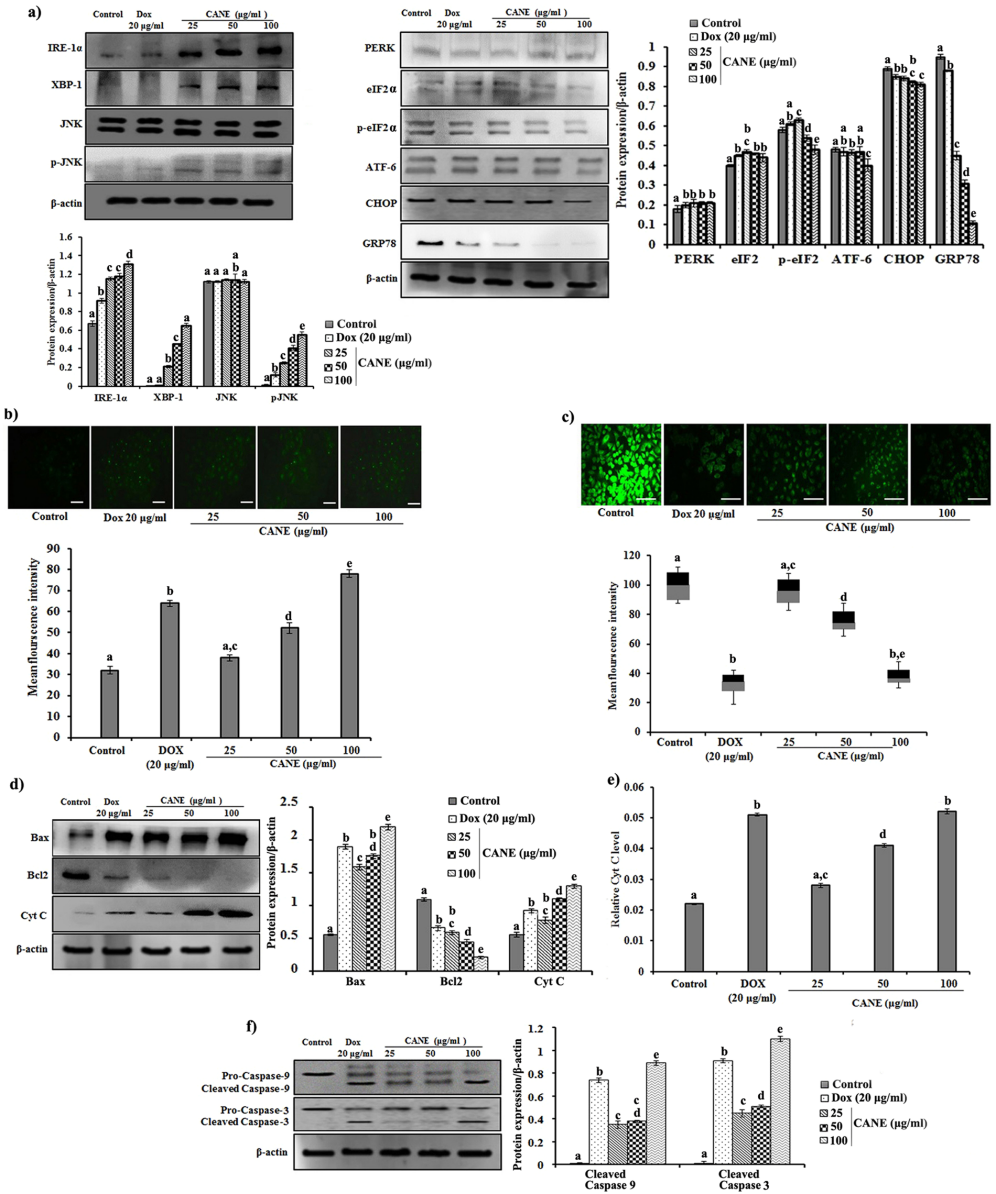
CANE sensitizes A549 cells to follow ER stress leads to mitochondria-mediated apoptotic pathway. (**a**) Western blotting of ER stress markers, β-actin used as loading control. Docetaxel (20 μg/ml) was used as a positive control while the surfactant-polysorbate 80 mix devoid of carvacrol was also used as a control. Densitometry analysis of IRE1-α, PERK, and ATF-6 was determined by Image J software. (**b**) Evaluation of intracellular Ca^+2^ accumulation. (**c**) Mitochondrial membrane potential of A549 cells treated with CANE (25-100 μg/ml). Fluorescent images were captured at 20X magnification [scale bar = 0.1 mm]. Image J software was used to determine the mean fluorescence intensity. (**d**) Effect of CANE on expression of Bax, Bcl2, and Cyt C in A549 cells. (**e**) Cyt C level in CANE-treated cells measured by spectrophotometric method. (**f**) Effect of CANE on expression of cleaved caspase-9, and 3 in A549 cells. Each value in the graph represents as the mean ± SD of three independent experiments. Values with different superscripts differ significantly from each other (*p* < 0.05).

ROS are known to induce stress and release of Ca^+2^ from ER. We observed a higher expression of IRE1-α in CANE treated cells, confirming its potential to induce ER stress (Fig. 4a). We also noticed a similar expression pattern for XBP-1, a downstream target of IRE1-α. In addition, higher expression of the pro-apoptotic marker, p-JNK was observed in CANE- treated cells (Fig. 4a). No effect of CANE was observed on the expression of the other ER stress markers PERK and ATF-6. However, down-regulation of CHOP, p-eIF2α, and GRP78 was observed in CANE-treated cells (Fig. 4a).

Further, fluorescent staining results revealed a dose-dependent increase of Ca^+2^ levels in CANE-treated cells. A 2.5 fold higher Ca^+2^ was observed at 100 μg/ml CANE treated cells as compare to the control signifying stress to ER (Fig. 4b).

### CANE induces apoptosis mediated by mitochondria

As depicted in Fig. 4c, CANE severely altered mitochondrial membrane potential (Δψm) in a dose-dependent manner. In addition, the immunoblot results confirmed the up- and down-regulation of pro-apoptotic (Bax) and anti-apoptotic (Bcl2) proteins, respectively, in CANE-treated cells as compared to the control (Fig. 4d). Moreover, an enhanced Cyt C release was observed in CANE-treated cells by both Western blotting and spectrophotometric methods (Fig. 4d,e). Further, we observed higher levels of cleaved caspase-9 and caspase-3 in cells treated with CANE in a dose-dependent manner (Fig. 4f).

### ROS scavenging results in A549 cell survival

**Figure 5:**
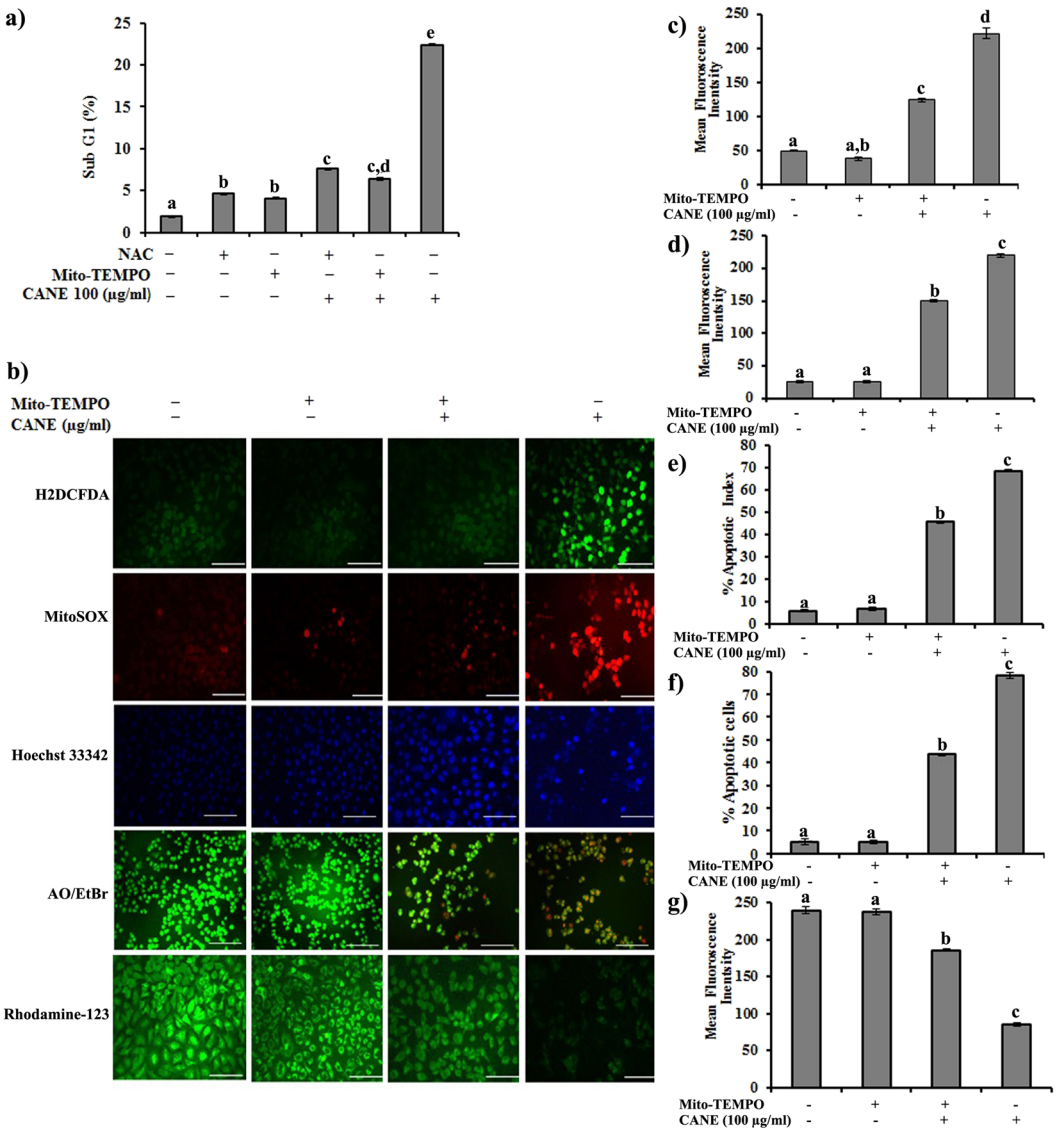
Inhibition of ROS production leads to A549 cell protection from apoptosis induced by CANE. (**a**) A549 cells were treated with the NAC, Mito-TEMPO, NAC + CANE (100 μg/ml), Mito-TEMPO + CANE (100 μg/ml) for 24 h. Apoptosis was analyzed as the sub-G1 (%) fraction by FACS analysis. (**b**) Fluorescent images representing apoptotic morphology, oxidative stress and mitochondrial membrane potential (Δψm). Fluorescent images were captured at 20X magnification [scale bar = 0.1 mm]. (**c**,**d**) Fluorescence intensity of H_2_DCFDA (green color) and MitoSOX (red color). (**e**) Apoptotic index based on Hoechst 33342 staining was calculated as the percentage of apoptotic nuclei compared to the total number of cells and is presented as the mean ± SD (n = 10). (**f**) Apoptotic cells based on AO/EtBr dual staining. (**g**) Fluorescent intensity of the cells representing mitochondrial membrane potential (Rhodamine 123). Image J software was used to determine the mean fluorescence intensity. Each value in the graphs represents as the mean ± SD of three independent experiments. Values with different superscripts differ significantly from each other (*p* < 0.05).

**Figure 6:**
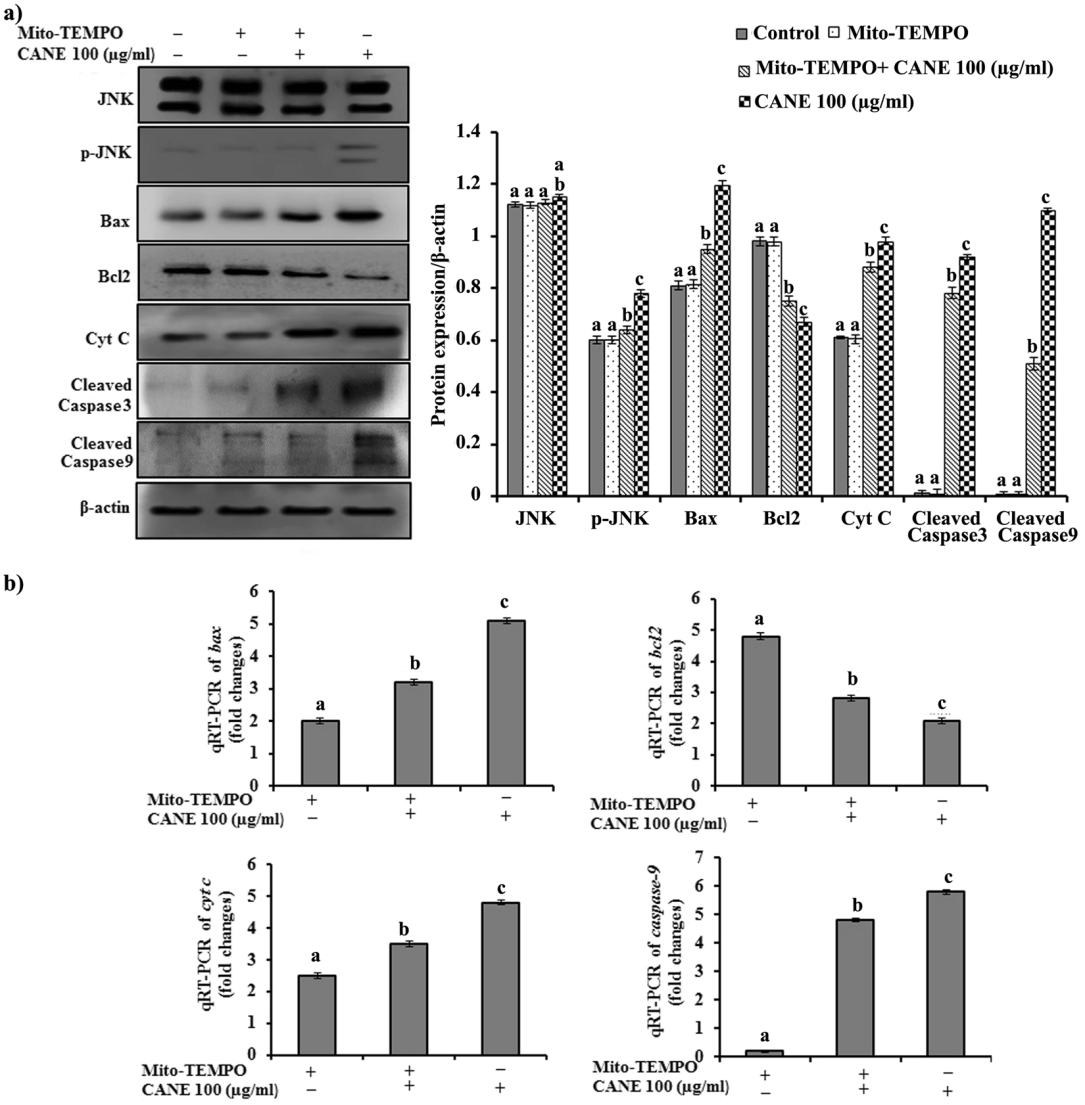
Effect of ROS inhibition using Mito-TEMPO on the vital apoptotic markers at translational and transcriptional level. (**a**) Cellular levels of protein markers JNK, p-JNK, Bax, Bcl2, Cyt C, caspase-3, caspase-9, and β- actin in A549 cells incubated with Mito-TEMPO (10 μM). Densitometry analysis of the respective proteins was evaluated by Image J software, and results were normalized with β-actin with respect to controls. (**b**) Quantitative RT-PCR (qRT-PCR) analysis of *bax*, *bcl2*, *cyt c*, *caspase-9*, and *β-actin* after treatment with CANE in conjunction with Mito-TEMPO. Mito-TEMPO controls expression of apoptotic genes at transcription level represented in fold change compared with control. Each value in the graphs represents as the mean ± SD of three independent experiments. Values with different superscripts differ significantly from each other (*p* < 0.05).

The results of the previous experiments ruminate that induction of ROS in A549 cells by CANE might be in the center of all the cellular events leading to apoptosis. To confirm this, A549 cells were first treated with N-acetyl-L-cysteine NAC (5 mM), a strong scavenger of ROS, prior to CANE (100 µg/ml) treatment and observed a marked reduction in ROS generation (Data not shown) and induction of apoptotic cell death by CANE (Fig. 5a). More precisely, we used Mito-TEMPO (10 µM), a specific antioxidant that selectively accumulates in mitochondria, prior to CANE (100 µg/ml) treatment. The success of ROS inhibition was confirmed by H_2_DCFDA and MitoSOX staining which suggested a reduced level of ROS and associated apoptotic morphological changes in the Mito-TEMPO + CANE-treated cells compared to only CANE-treated cells (Fig. 5b). Likewise, FACS results displayed a decreased cytotoxic potential of CANE in the presence of Mito-TEMPO (Fig. 5a). Moreover, Mito-TEMPO- treated cells were capable to restore the alter Δψm induced by CANE (Fig. 5b). The immunoblot results confirmed reduced levels of the apoptotic markers including Bax, p-JNK, Cyt C, and caspase-3, 9, and increased level of anti-apoptotic Bcl2 in Mito-TEMPO + CANE treated cells as compared to only CANE treated cells (Fig. 6a).

### Mito-TEMPO reverses CANE-induced mRNA transcription of *bax*, *bcl2*, *cyt c* and caspase -9

Effect of Mito-TEMPO on CANE-mediated expression of *bax*, *bcl2*, *cyt c*, and *caspase-9* in A549 cells at the transcriptional level was evaluated by qRT-PCR. CANE induced upregulation of *bax*, *cyt c*, and *caspase-9*, and down-regulation of *bcl2* were abolished by Mito-TEMPO, suggesting the involvement of ROS as the key regulators of apoptosis (Fig. 6b).

### Mito-TEMPO blocks CANE induced Bax and cytochrome C translocation

**Figure 7:**
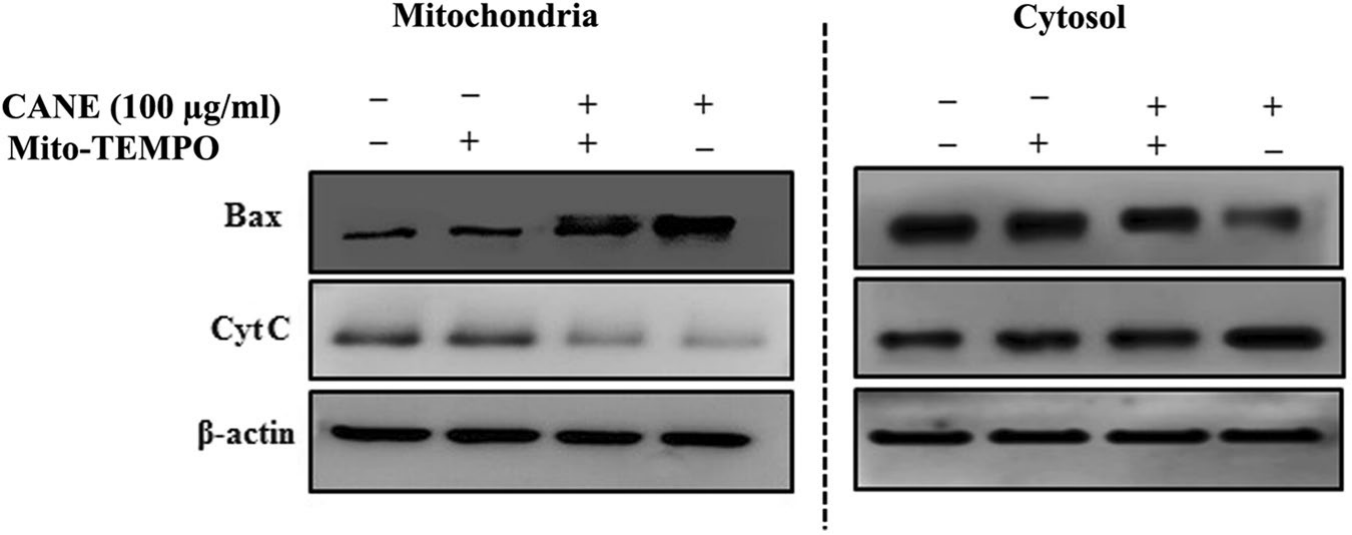
Mito-TEMPO prevents translocation of Bax and cytochrome C. Translocation of Bax from the cytosol to mitochondria and release of Cyt C from mitochondria in A549 cells incubated with Mito-TEMPO (10 μM).

Translocation of Bax from the cytosol to mitochondria and release of Cyt C from mitochondria are key events during mitochondrial-mediated apoptosis. A549 cells treated with Mito-TEMPO + CANE effectively blocked translocation of Bax and release of Cyt C, unlike the only CANE-treated cells (Fig. 7).

### Apoptotic potential of CANE against PC-9 cells

**Figure 8:**
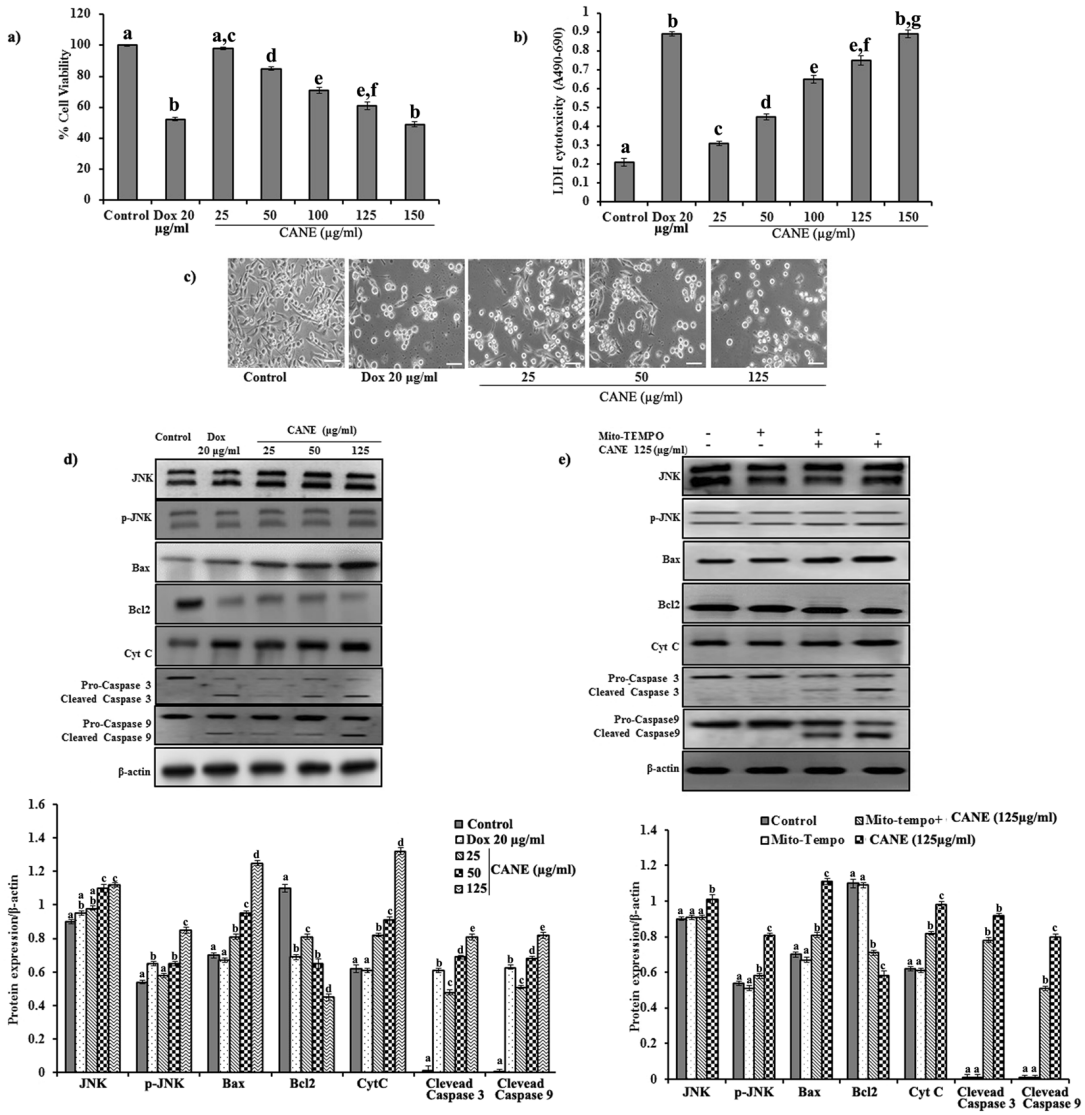
Apoptotic potential of CANE against PC-9 cells. (**a**,**b**) MTT and LDH assay, respectively, of CANE (25–150 μg/ml)-treated PC-9 cells after 24 h of incubation. Docetaxel (20 μg/ml) was used as a positive control while the surfactant-polysorbate 80 mix devoid of carvacrol was also used as a control. (**c**) Morphological changes in PC-9 cells after treatment with CANE (25–125 μg/ml). Images were captured at 20X magnification [scale bar = 0.1 mm]. (**d**) Effect of CANE on expression of JNK, Bax, Bcl2, Cyt C, caspase-9, and 3 in PC-9 cells. (**e**) Cellular levels of protein markers JNK, p-JNK, Bax, Bcl2, Cyt C, caspase-3, caspase-9, and β- actin in PC-9 cells incubated with Mito-TEMPO (10 μM). Densitometry analysis of the respective proteins was evaluated by Image J software, and results were normalized with β-actin with respect to controls. Each value in the bar graph represents the mean ± SD of three independent experiments. Values with different superscripts differ significantly from each other (*p* < 0.05).

As depicted in the Fig. 7, CANE exhibited a dose-dependent cytotoxicity with 62.1 and 52.2% cell viability at 125 and 150 μg/ml concentrations, respectively (Fig. 8a). In addition, western blotting results support the mitochondrial mediated apoptotic potential of the CANE by the involvement of Cyt C, caspase-3 and 9 (Fig. 8d). Scavenging of ROS using mitochondrial specific antioxidant Mito-TEMPO (10 µM) reverted the apoptotic potential of CANE, signifying its role in the induction of oxidative stress inside the cell (Fig. 8e).

### CANE reduces tumor formation in mice

**Figure 9:**
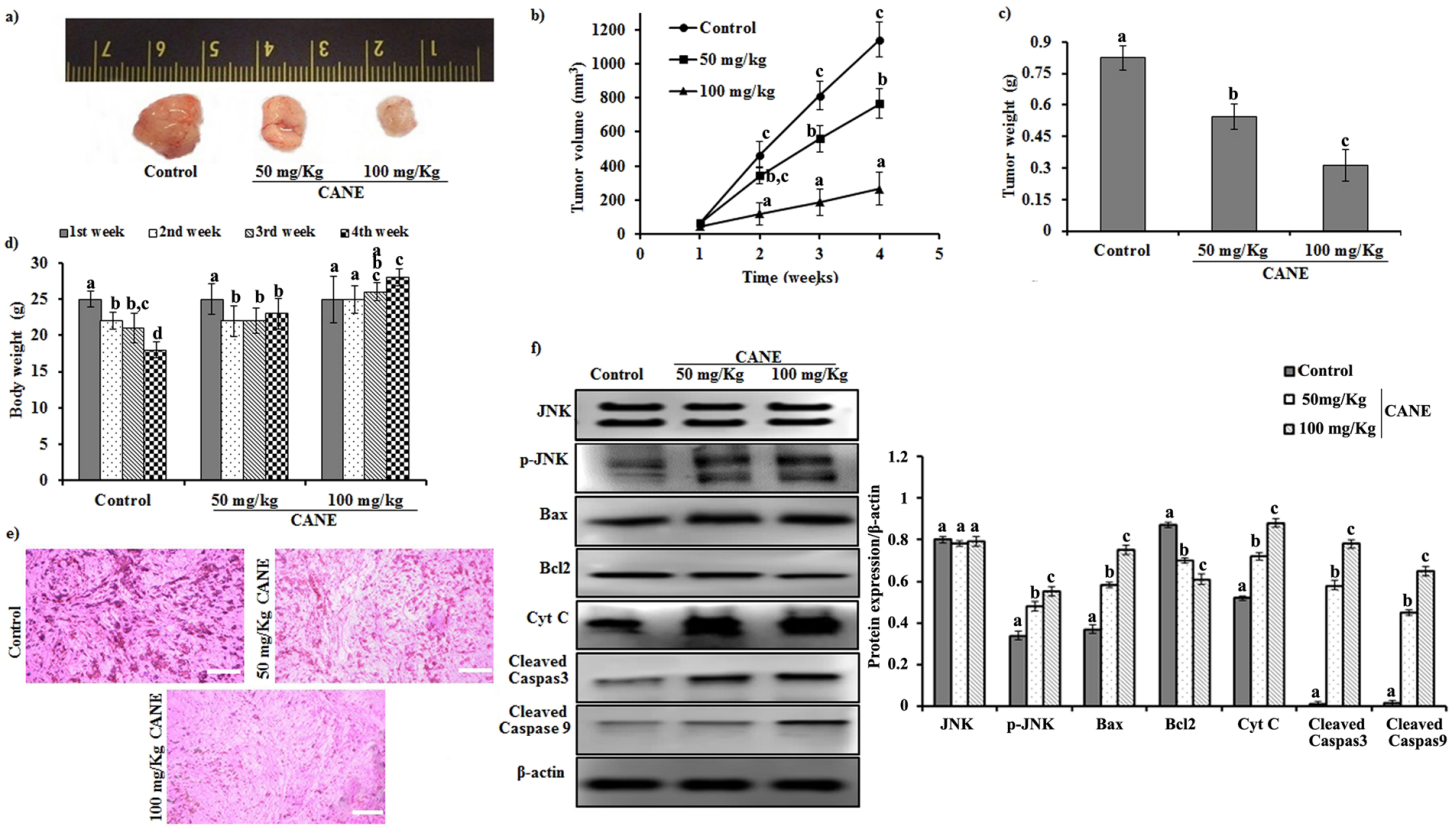
CANE inhibits tumor growth. (**a**) Tumor images excised from different groups of mice after 4 weeks. (**b**) Tumor volume with respect to time. (**c**) Tumor weight after 4 weeks. (**d**) Body weight with respect to time. (**e**) Histological images of tumor observed under light microscope. Images were captured at 40X magnification [scale bar = 0.05 mm]. (**f**) Expression of JNK, p-JNK, Bax, Bcl2, Cyt C, caspase-3, caspase-9, and β- actin in tumor tissue. Each value in the bar graph represents the mean ± SD of three independent experiments. Values with different superscripts differ significantly from each other (*p* < 0.05).

Results demonstrated a strong antitumor potential of CANE. As depicted in Fig. 9, CANE effectively reduced the tumor growth in a dose-dependent manner. A significant (*p* < 0.05) 34.2 and 62.1% reduction in tumor weight was observed in the mice treated with 50 and 100 mg/Kg CANE, respectively as compared to the control (Fig. 9c). On the other side, body weights reduced constantly with the progression of time in the control mice, however, a lower change was observed in the 50 mg/kg CANE treated mice. In contrast, body weights of 100 mg/kg CANE treated mice remained static up to the second week and increased further up to 4 weeks (Fig. 9d).

Histopathological study of the tumor revealed the apoptotic features in the CANE treated mice as evident by fragmented and condensed nuclear morphology unlike to small, densely packed cells with slight invasion to the surrounding tissue in the control group (Fig. 9e). Moreover, immunoblotting results suggested induction of apoptosis in the CANE treated groups as noticed by higher expression of p-JNK likewise up- and down-regulation of Bax and Bcl2 as compared to vehicle control. Besides, higher expressions of Cyt C and associated caspase-9 and 3 were observed in response to CANE signifying its apoptotic role in the tumor (Fig. 9f).

## Discussion

Carvacrol is the monoterpene phenolic compound with numerous biological activities^10,11^. Despite its many health benefits, carvacrol is highly affected by light, air, pressure and heat. Moreover, its volatile nature reduces its shelf-life and acts as the principal limiting factor for its industrial use^21^. Encapsulation is a way to enhance the shelf-life from the physical and chemical agents without altering their biological properties^21^. In the past few years, NEs have gained more attention due to their unique physicochemical and functional properties^22^. Therefore, the present study commenced to formulate CANE by an energy intensive ultrasonication method. NEs can be prepared by both low and high energy techniques through dispersion of one liquid phase into another immiscible liquid phase. Among energy intensive methods, ultrasonication consider as simple, cost-effective, clean and prompt aseptic technique^16^, wherein large droplets ruptured into small droplets by ultrasound leading to the formation of nano-scale droplets^19,23^. NEs are stabilized by the addition of surfactants, which reduce interfacial tension between oil and water phases. The tendency of surfactants to disperse into two immiscible phases is referred to as hydrophilic-lipophilic balance (HLB)^23^, and HLB greater than 10 is more favorable for O/W emulsion^24^. We selected polysorbate 80 as a surfactant (HLB, 15), which is regarded as safe for using in pharmaceutical and food industries^16^. During NE formulation, we observed an inverse relationship between volumes of polysorbate 80 with formation of droplet size. We speculate that the reduction in size is associated with the increase in interfacial area and the decrease in interfacial tension by surfactant, which is in accordance with previous finding, suggesting the similar relationship between droplet size and surfactant volume^16^. Role of surfactants on the adjustment of interfacial tension has been well mentioned in the literature^18,25^.

It has been reported that the net surface charge of a NE has a direct effect on enhancing drug membrane permeability, stability, and efficacy^19,26^. We observed a negative surface charge on the nano-droplets, which increased in the emulsion with increasing surfactant concentration, confirming the stability of the emulsion due to sufficient electrostatic repulsion and thus prevention of droplet aggregation. The findings corroborate with earlier published reports where higher electrostatic repulsion inhibit droplet aggregation^27,28^.

Finally, we confirmed the size and morphology of the CANE by electron microscopy, which is a well adopted method for nano-droplet evaluation together with DLS^29,30^ and observed a slight change in size that obtained from DLS analysis.

There are various cell death mechanisms such as apoptosis^31^, necrosis^32^, and autophagy^33^ by which a drug affects with the cancer cells. Among them, apoptosis is the most common way to kill the cancer cells. Thus, induction of apoptosis by a drug emerged as a potential way to eradicate the cancer cells. Numerous published reports suggest the anticancer potential of monoterpenes including carvacrol against various cell lines by the induction of apoptosis^14^, however, there are limited studies showing the anticancer potential of NEs derived from essential oil and more specifically CANE.

Determination of cytotoxicity is the first and foremost important step to examine the apoptotic potential of any drug. We observed reduced cell viability in CANE-treated cells by MTT and LDH assays, which was well supported by FACS and clonogenic assay. Results are in acquaintance with previous studies suggesting the cytotoxic potential of carvacrol against various cancer cell lines^13,34^. To the best of our knowledge, there has been no study so far suggesting the cytotoxic effect of CANE, though there are many studies suggesting the antimicrobial potential of carvacrol NE^35–37^.

Nuclear fragmentation and chromatin condensation are the hallmarks of apoptosis. We observed increased chromatin condensation and pycnonuclei formation by Hoechst staining, which is consistent with AO/EtBr dual staining signifying apoptosis in CANE-treated cells. There are only limited numbers of studies representing the anticancer potential of NEs derived from essential oil. Though in a study done on NE made up of essential oil of *N*. *sativa* elucidated cytological changes such as chromatin condensation leading to apoptotic cell death against human breast cancer (MCF-7) cells^16^.

Accumulating reports suggest a direct role of ROS in DNA damage, leading to apoptosis^15,38,39^. We also observed the potential role of CANE in inducing ROS production, both in the cytosol and mitochondria, which provoked many cellular events leading to apoptosis. Results are consistent with published reports suggesting ROS induction in the presence of various essential oil^40^ and their principal components^41,42^, including carvacrol^13,34^. However, only few studies are available on the effect of NEs derived from these essential oils on the ROS generation.

Mitochondria is the major site of intracellular ROS production^43^ which leads to mitochondrial dysfunction and loss of Δψm as a result of opening mitochondrial permeability transition pore (MPTP) that provide passage to release Cyt C^44^. We detected a marked difference in Δψm and associated release of Cyt C in response to CANE that ultimately triggered the caspase cascade by activating caspase-3, which plays a central role in the progression of apoptosis by activation of many important cellular proteins, including endonucleases^44,45^.

Mitochondria and ER remain in the close proximity and release of ROS from the mitochondria instigating subsequent stress on ER, a site for protein translocation, folding and posttranslational modification, and highly sensitive to stress^46,47^. During the disturbing environment, protein folding is highly affected thus starts to accumulate in ER lumen and creates a condition known as ER stress. To overcome such conditions, cells stimulate many cellular events collectively known as the unfolded protein response (UPR), however, a prolonged ER stress became cytotoxic leading to apoptotic cell death^48^. ER stress is associated with the three major signaling proteins IRE1-α, PERK, and ATF-6^47^. Among them, IRE1-α also known to be involved in cell death whereas, PERK-eIF2 involved in cell survival^15^. IRE1-α is a principal signaling protein with serine threonine kinase domain, and an endoribonuclease domain which it uses to splice XBP-1 mRNA and activate XBP-1, an important protein during ER stress^47^. We observed up-regulation of IRE1-α and its downstream target XBP-1 in response to CANE, suggesting its potential role in ER stress. IRE1-α is also associated with the activation of ASK1, which stimulates activation of the Jun-N-terminal kinase (JNK) and p38 MAPK to promote apoptosis^46^. Also, we observed high protein expression of p-JNK in connection with up-regulation of IRE1-α stimulated by CANE^46^.

The lumen of ER is the major storehouse of Ca^+2^, which plays a critical role in many physiological events^49^ along with the proper function of ER-associated chaperons^46^. Hence, any imbalance in ER Ca^+2^ levels has severe effects on ER function, leading to stress. ROS are known to induce Ca^+2^ release from the ER and also inhibit the intracellular Ca^+2^ pump for its clearance from the cytosol, as a result, starts to accumulate in mitochondria and influence on Δψm^43^. We observed a high Ca^+2^ level in CANE-treated cells responsible for ER stress and altering Δψm.

CANE induced elevation of IRE1-α levels mediating JNK activation, which caused Bax translocation from the cytosol to mitochondria and opened the MPTP, facilitating Cyt C releases that activated proteases such as caspase-9 and 3. Our earlier results suggest that CANE efficiently induced ROS and generated oxidative stress in A549 cells, leading to apoptosis. To prove this, we blocked ROS using NAC and observed diminishing effect of CANE and confirmed that ROS is the main agent behind CANE-mediated cytotoxicity. More specifically, to locate the site of ROS production, we used the mitochondria-specific antioxidant Mito-TEMPO and observed the prevention of CANE-induced ROS generation and collapse of Δψm. The apoptotic protein levels were reverted to normal levels, and apoptosis was diminished in the Mito-TEMPO treated group. Also, we found an impact on Bax and Cyt C translocation in the presence of Mito-TEMPO. These results are consistent with the previous finding wherein Mito-TEMPO inhibited ROS production and associated Ca^+2^ imbalance and maintained Δψm to prevent mitochondrial-mediated necrosis and apoptosis along with translocation of Bax^43^. Our findings are also supported by the previous study which demonstrated consequences of ROS scavenger against doxorubicin-induced apoptosis by altering the Bax, Cyt C translocation and caspase activation^50^.

To evaluate the broad spectrum anticancer potential of CANE against different lung cancer cell line, we tested it against human lung adenocarcinoma PC-9 cells and observed a dose-dependent cytotoxicity. However, unlike to A549 cells, CANE displayed a high IC_50_ value against PC-9 cells. In the presence of Mito-TEMPO, apoptotic potential of CANE diminished as evident by altered protein expression of Bax, Bcl2 Cyt C, caspase- 3 and 9 implying the involvement of mitochondrial ROS as a major event in connection with CANE mediated cytotoxicity.

This study confirmed a strong cytotoxicity of CANE *in vitro;* hence, we moved ahead and evaluated its antitumor activity (*in vivo*). Our *in vivo* results are consistent with the *in vitro* results signifying the strong potential of CANE to regulate tumor growth via induction of apoptosis. The results collectively propose the key role of CANE in mitochondrial ROS generation, which initiated numerous cellular events leading to mitochondrial-mediated cell death. Additionally, antitumor potential of CANE suggested it to be as a promising candidate against lung cancer.

## Conclusions

Based on the findings, we conclude that CANE principally induced production of ROS inside mitochondria of A549 cells, which provoked many cellular events leading to mitochondria-mediated intrinsic apoptosis. To the best of our knowledge, this is the first study in which CANE exhibited substantial apoptotic potential against human lung adenocarcinoma A549 cells. Owing the good anticancer potential of CANE, we propose it as a suitable drug for lung cancer therapy after sufficient clinical trials.

## Materials and Methods

### Chemicals, antibodies and reagents

All chemicals, unless otherwise stated were of the highest quality and used as supplied. Carvacrol (99.9%) and polysorbate 80 were purchased from the Sigma-Aldrich (St. Louis, USA). Primary antibodies against β-actin, PERK, GRP78, IRE1-α, ATF-6, XBP-1, eIF2α, p-eIF2α, JNK, p-JNK, caspase-3, caspase-9, Bax, Bcl2, and cytochrome C as well as horseradish peroxidase (HRP)-conjugated secondary antibody were purchased from Santa Cruz Biotechnology, Inc. (Santa Cruz, CA, USA).

### Cell lines and cell culture

A549, PC-9, a human lung adenocarcinoma, and normal bronchial epithelium cells (BEAS-2B) were purchased from the American Type Culture Collection (Manassas, VA), and cultured in RPMI-1640 (Invitrogen, Carlsbad, CA) media, supplemented with 10% (v/v) fetal calf serum (Invitrogen, Carlsbad, CA) and 1% penicillin-streptomycin cocktail at 37 °C in 5% CO_2_ incubator.

### Preparation of carvacrol nanoemulsion

Oil in water (O/W) nanoemulsion (NE) was prepared by adding carvacrol (1.5%), non-ionic surfactant, and emulsifier polysorbate 80 in water under constant shaking conditions. NE was formulated in aqueous medium by adding carvacrol with polysorbate 80 to obtain 1:1, 1:2 and 1:3 (v/v) proportions. Finally, all three formations were sonicated at 25 khz in a sonicator with 750 W for 10 min. Tubes were kept in an ice bucket throughout the sonication period to neutralize the deleterious effect of generated heat.

### Characterization of nanoemulsion

Droplet size, polydispersity index (PDI) and zeta potential of the formulated CANEs were analyzed by dynamic light scattering (DLS) analysis using a zeta-potential and particle size analyzer (ELSZ-2000, Otsuka Electronics Co., Ltd. Japan). The mean droplet sizes of CANEs was determined using software generated intensity, volume, and number of distributions. The morphology of CANE was determined by scanning electron microscope (SEM).

### Cytotoxicity and morphological assessment

The cytotoxic effect of CANE was first determined by MTT assay. Briefly, 5 × 10^4^ A549 cells/well were incubated in the presence of CANE at the concentrations of 25, 50, 100, and 150 μg/ml for 24 h at 37 °C in a CO_2_ incubator. After incubation, cells were treated with MTT solution (5 mg/ml) to produce dark blue colored formazan crystals, which were further dissolved in 50 μl of DMSO. Finally, absorbance was measured at 540 nm in a microplate reader (Bio-Tek instrument Co., WA, USA).

Cytotoxicity was also determined by the lactate dehydrogenase (LDH) assay. For the LDH assay, cells were treated with various concentrations of CANE, as mentioned above, for 24 h at 37 °C. After incubation, media were removed and processed to evaluate extracellular LDH release using an LDH detection kit (Sigma-Aldrich, St.Louis, USA) as per the manufacturer’s guidelines. Finally, cytotoxicity was evaluated by calculating absorbance at 490 and 690 nm.

Furthermore, microscopic examination was done to determine morphological changes in A549 cells after exposure to CANE (25, 50 and 100 μg/ml) using an inverted microscope (Nikon Eclipse TS200, Nikon Corp., Tokyo, Japan). In addition, cytotoxic potential of CANE against PC-9 cells was evaluated by MTT, LDH assay as mentioned earlier along with the western blotting of the apoptotic proteins.

### Clonogenic assay

A total of 5 × 10^4^ cells/well were seeded in a 12-well culture plate and allowed to attach for a period of 24 h, after which CANE (25, 50, and 100 μg/ml) treatment was administered and cells were incubated for 7 days at 37 °C in a CO_2_ incubator. Finally, cells were washed with PBS and stained with 0.5% crystal violet.

### Flow cytometry analysis

Control (surfactant-polysorbate 80 mix devoid of carvacrol) and CANE-treated cells were suspended in 100 µl (PBS), followed by the addition of 200 µl of ethanol (95%) and incubated at 4 °C for 1 h. Afterward, cells were washed with PBS and suspended in 250 µl of sodium citrate buffer (pH 8.4) containing RNase (12.5 µg) for 30 min. Finally, cellular DNA was stained with propidium iodide solution (50 µg/ml), and stained cells were analyzed using fluorescent-activated cell sorting with a flow cytometer (ThermoFisher, Korea). Relative DNA content was determined based on relative red fluorescent intensity.

### Determination of apoptotic morphological changes

Nuclear fragmentation was evaluated by Hoechst staining. Briefly, cells were seeded in a 12-well-plate and allowed to attach for 24 h, followed by CANE (25, 50, and 100 μg/ml) treatment for an additional 24 h. After incubation, cells were stained with Hoechst (1 µg/ml), and images were captured under an EPI fluorescence microscope (Nikon, Japan). In addition to Hoechst staining, acridine orange (AO) and ethidium bromide (EtBr) staining were carried out to detect apoptotic cell formation. Briefly, attached cells were treated with different concentrations of CANE for 24 h, followed by staining with AO/EtBr (1:1). Finally, fluorescence images were captured under an EPI fluorescence microscope (Nikon, Japan).

### Determination of intracellular ROS and mitochondrial superoxide levels

Intracellular reactive oxygen species (ROS) production in response to CANE was determined using H_2_DCFDA fluorescent stain. Briefly, cells were seeded in a 12-well culture plate and allowed to attach for 24 h, followed by CANE (25, 50, and 100 μg/ml) treatment. After 24 h of treatment, cells were washed with PBS and subsequently incubated with 20 µM H_2_DCFDA dye for 30 min. Cells were washed twice with PBS, and ROS formation was immediately detected using an EPI fluorescence microscope (Nikon, Japan). Fluorescent intensity was quantified by using Image-J software.

The potential effect of CANE on production of mitochondrial superoxide was evaluated by staining the cells with MitoSOX Red, a mitochondrial superoxide indicator. Briefly, attached cells were treated with different concentrations of CANE for 24 h. After incubation, cells were washed twice with PBS and stained with 5 μM MitoSOX Red for 10 min. Finally, fluorescent images were captured using an EPI fluorescence microscope (Nikon, Japan). Mean fluorescence intensity was quantified using Image-J software.

### Measurement of intracellular calcium ion (Ca^+2^)

A549 cells were grown in a 6-well culture plate and treated with various concentrations of CANE (25, 50, and 100 µg/ml). After 24 h of incubation, cells were washed twice with PBS and stained with 5 μM fura-2 AM for 60 min. Finally, cells were washed three times with HEPES buffer and visualized under an EPI fluorescence microscope (Nikon, Japan). The mean fluorescent intensity of fluorescent images was quantified by Image-J software.

### Measurements of mitochondrial membrane potential (Δψm)

A549 cells were grown in a 12-well culture plate and treated with various concentrations of CANE (25, 50, and 100 µg/ml). After 24 h of incubation, cells were washed with PBS and stained with rhodamine-123 (1 μg/ml) for 30 min. Finally, fluorescent images were analyzed under an EPI fluorescence microscope (Nikon, Japan).

### Determination of cytochrome C release

Cytosolic cytochrome C (Cyt C) level was evaluated by a spectrophotometric method^51^. In brief, attached A549 cells were treated with various concentrations of CANE (25, 50, and 100 µg/ml) over 24 h. Cells were then homogenized with buffer (50 mM Tris, 2 mM EDTA, 1 mM phenylmethylsulfonylfluoride, pH 7.5) in the presence of 2% glucose and centrifuged at 2,500 rpm for 10 min. Obtained supernatant was mixed with 0.5 g/ml of ascorbic acid, and absorbance was measured at 550 nm.

### Immunoblot analysis

A549 cells were treated with various concentrations of CANE (25, 50, and 100 µg/ml) for 24 h, followed by protein isolation using RIPA lysis buffer. An equal amount of protein lysate (50 μg in each lane) was separated in reducing polyacrylamide gel and further transferred into a polyvinyldenefluoride (PVDF) membrane (Roche Diagnostics, Indianapolis, IN, USA) by electroblotting. The membrane was subsequently probed with appropriate primary antibodies, followed by horseradish peroxidase (HRP)-conjugated secondary antibody, and visualized by enhanced chemiluminescence (ECL) according to the recommended procedure (Amersham Pharmacia, Piscataway, New Jersey).

### Isolation of mitochondrial proteins

Mitochondrial proteins were isolated from A549 cells by treatment with mitochondrial extraction buffer (70 mM sucrose, 200 mM mannitol, 10 mM HEPES, 1 mM EGTA), followed by homogenization using a Dounce homogenizer. The cell suspension was centrifuged at 600xg, and the obtained supernatant was re-centrifuged at 11,000xg to separate mitochondria from cytosolic proteins. Quantification of proteins was carried out by the Bradford method^52^.

### Isolation of RNA and quantitative real-time PCR (qRT-PCR)

Total RNA was isolated from A549 cells using an RNA-spinTM extraction kit (Intron Biotechnology, Korea) as per the manufacturer’s instructions and quantified by a Qubit22s 2.0 fluorometer RNA assay kit (Life technologies, USA). cDNA synthesis was carried out using a Maxime RT Premix cDNA synthesis kit (Intron Biotechnology, Korea) according to the manufacturer’s instructions. Finally, qRT-PCR was performed using an Agilent technology qPCR system (CA, USA). Following PCR primers were used for *bax* (F, 5′-CTGCAGAGGATGATTGCCG-3′, R, 5′-TGCCACTCGGAAAAAGACCT-3′), *bcl2* (F, 5′-TCCCTCGCTGCACAAATACTC-3, R, 5′-ACGACCCGATGGCCATAGA-3′), *cyt c* (F, 5′-CCAGTGCCACACCGTTGAA-3′, R, 5′-TCCCCAGATGATGCCTTTGTT-3′), *caspase-9* (F, 5′-CGAACTAACAGGCAAGCAGC-3′, R, 5′-ACCTCACCAAATCCTCCAGAA C-3′), and *β-actin* (F, 5′-AACTACCTTCAACTCCATCA-3′, R, 5′-GAGCAATGATCTTGATCTTCA-3).

### *In vivo* animal studies

The antitumor potential of CANE was evaluated by inducing tumor in nude mice by xenografting of A549 cells. The tumor was induced by injecting A549 cells (1 × 10^6^) subcutaneously to 4 week old male nude mice. Once the tumor reached to 50 mm^3^ size the mice were randomly divided into three groups (each group with four mice). Group I considered as vehicle control (surfactant-polysorbate 80 mix devoid of carvacrol) while the group II and III were treated with CANE 50 and 100 mg/kg body weight (BW), respectively. Treatment of CANE was given orally three times in a week up to four weeks. Tumor volume and body weight in all the three groups were measured every week. Tumor volume was calculated using the equation [(width^2^ × length)/2]. At the end of fourth week, mice were sacrificed and the tumor was removed for evaluation of immunoblotting, tumor weight and histopathological examination.

### Hematoxylin and eosin (H & E) staining

The tumor was stored in 10% formalin for 48 h. Finally, tissue was dehydrated and embedded in paraffin followed by sectioning and H and E staining. Histopathological changes were observed by microscopic examination (Nikon, Japan).

### Statistical analysis

All experiments were carried out in triplicates. The results were expressed as mean ± standard deviation (SD) of three independent experiments. Multiple comparisons were performed using one-way ANOVA followed by Duncan test for post hoc analysis using SPSS16 software. *p* values < 0.05 were considered statistically significant.

### Data availability

The authors declare that all the other data supporting the finding of this study are available within the article and from the corresponding author on reasonable request.

### Ethics approval and consent to participate

Animal experimental work conducted in the present study was in accordance with the rules and regulation of animal ethical committee of Daegu University, Korea (Approvl # DGU000011365) for the care and use of laboratory animals.

### Manuscript comment

Pictures or photo structures as shown in the figures were taken by one of the authors Ashutosh Bahuguna as experimental data. We have not taken or adopted any picture of figure from any other sources.

## Electronic supplementary material

DataSet 1
