# Supplementary material for: In vitro and in vivo antitumor potential of carvacrol nanoemulsion against human lung adenocarcinoma A549 cells via mitochondrial mediated apoptosis
Source: Sci Rep. 2018 Jan 9;8:144. doi: 10.1038/s41598-017-18644-9 (PMC5760660; doi:10.1038/s41598-017-18644-9)
Supplement: Supplementary file 1 — DataSet 1 [file 41598_2017_18644_MOESM1_ESM.doc]

**Supplementary Data Set 1**

***In vitro* and *in vivo* antitumor potential of carvacrol nanoemulsion against human lung adenocarcinoma A549 cells via mitochondrial mediated apoptosis**

**Imran Khan^1^,**^¶^**, Ashutosh Bahuguna^1^,**^¶^**, Pradeep Kumar^2,*^, Vivek K. Bajpai^3,*^, Sun Chul Kang^1,*^**

^1^Department of Biotechnology, Daegu University, **Gyeongsan, Gyeongbuk** 712-714, Republic of Korea

^2^Department of Forestry, North Eastern Regional Institute of Science and Technology, Nirjuli, India

^3^Department of Energy and Materials Engineering, Dongguk University-Seoul, Seoul, Republic of Korea

**^¶^These authors contributed equally to this work**

***Correspondence:**

**Prof. (Dr.) Sun Chul Kang;** E-mail: sckang@daegu.ac.kr

**Co-correspondence:**

**Dr.** **Pradeep Kumar;** E-mail: pkbiotech@gmail.com

**Dr. Vivek K. Bajpai;** E-mail: vbajpai04@yahoo.com

**Control Dox (20 ) 25 50 100**

**μg/ml**

**CANE μg/ml**

**p-JNK**


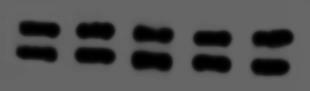


**JNK**


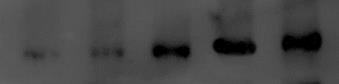


**IRE-1α**


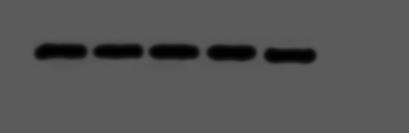


**β-actin**


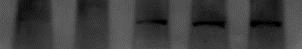


**XBP-1**


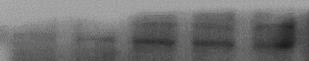


**Figure S4a. Figure 4d. CANE sensitizes A549 cells to follow ER stress leads to mitochondria-mediated apoptotic pathway.** Western blotting of ER stress markers.

**Control Dox (20) 25 50 100**

**μg/ml**

**CANE μg/ml**

**PERK**


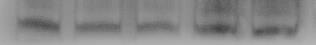

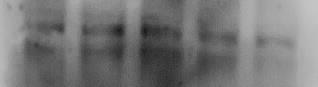


**CHOP**


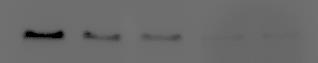


**GRP78**

**eif2**


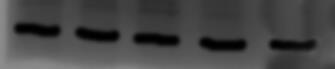


**β-actin**

**ATF-6**


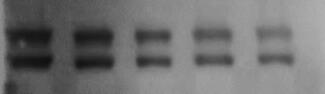


**p-eIF2**


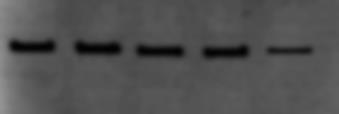

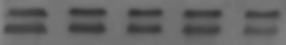


**Figure S4a. Figure 4d. CANE sensitizes A549 cells to follow ER stress leads to mitochondria-mediated apoptotic pathway.** Western blotting of ER stress markers.

**Control Dox (20) 25 50 100**

**μg/ml**

**CANE μg/ml**

**Bax**


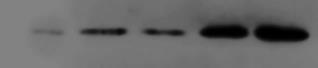


**Cyt C**


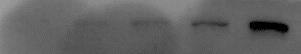


**Bcl2**


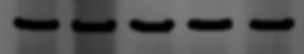


**β-actin**


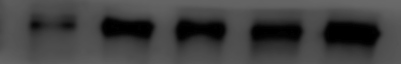


**Figure S4d. CANE sensitizes A549 cells to follow ER stress leads to mitochondria-mediated apoptotic pathway.** Effect of CANE on expression of Bax, Bcl2, and cyt c in A549 cells.

**Control Dox (20) 25 50 100 μg/ml**

**CANE μg/ml**

**Pro-Caspase-9**

**Cleaved Caspase -9**


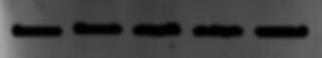


**β-actin**


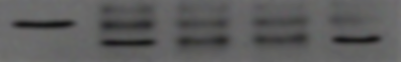

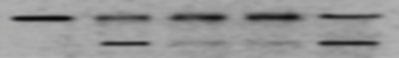


**Pro-Caspase-3**

**Cleaved Caspase -9**

**Figure S4f. CANE sensitizes A549 cells to follow ER stress leads to mitochondria-mediated apoptotic pathway.** Effect of CANE on expression of cleaved caspase-9, and 3 in A549 cells.

**CANE (100** **μg/ml) - - + +**

**(Mito-TEMPO) - + + -**

**Cleaved Caspase -9**


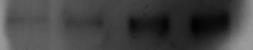


**Cleaved Caspase -3**


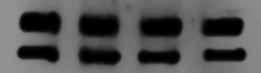


**JNK**


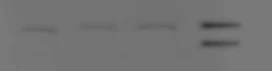


**p-JNK**

**BAX**

**BCl2**

**β-actin**

**Cyto C**


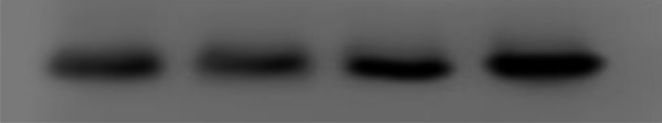

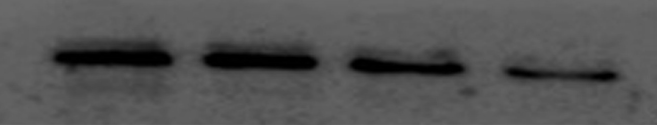

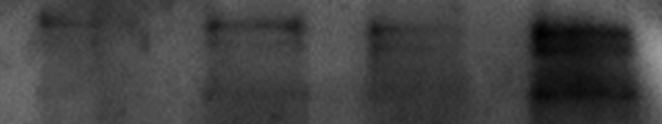

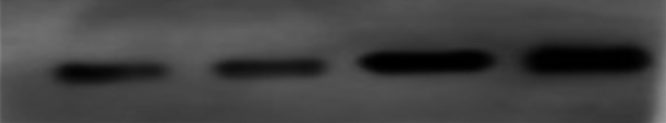

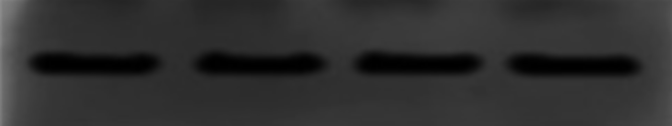


**Figure S6a. Effect of ROS inhibition using Mito-TEMPO on the vital apoptotic markers at translational and transcriptional level.** cellular levels of protein markers JNK, p-JNK, Bax, Bcl2, Cyt C, caspase-3, caspase-9, and β- actin in A549 cells incubated with Mito-TEMPO.


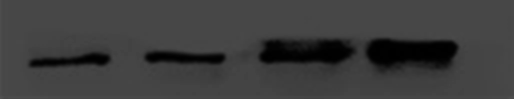

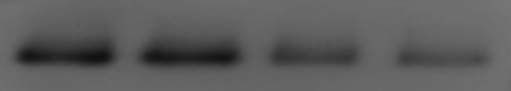

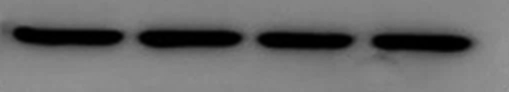

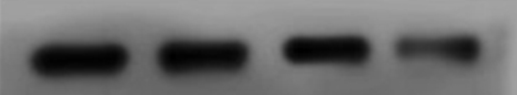

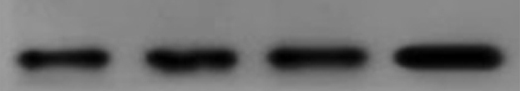

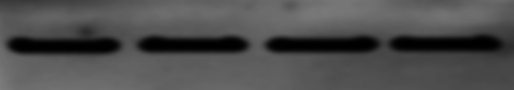


**β-actin**

**Bax**

**Cyto C**

**CANE ( 100 μg/ml) - - + +**

**Mito-TEMPO - + + -**

- **- + +**
- **+ + -**

**Figure S7. Mito-TEMPO prevents translocation of Bax and cytochrome C.** Translocation of Bax from the cytosol to mitochondria and release of Cyt C from mitochondria in A549 cells incubated with Mito-TEMPO.

**Control Dox (20) 25 50 125**

**CANE μg/ml**


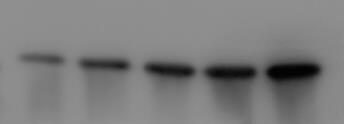


**Bax**

**JNK**


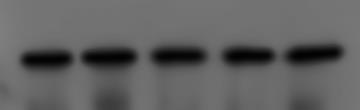


**β-actin**

**p-JNK**

**Bcl2**

**Cyt C**

**Pr- Caspase 3**

**Cl. Caspase 3**

**Pr- Caspase 9**

**Cl. Caspase 9**


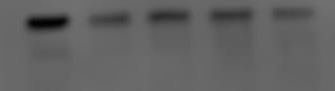

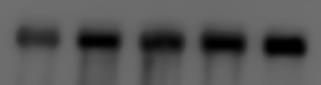

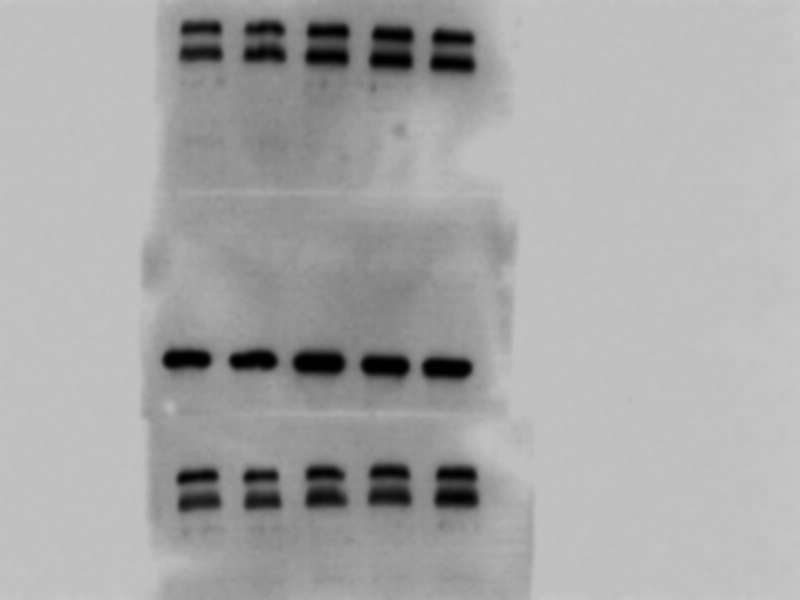

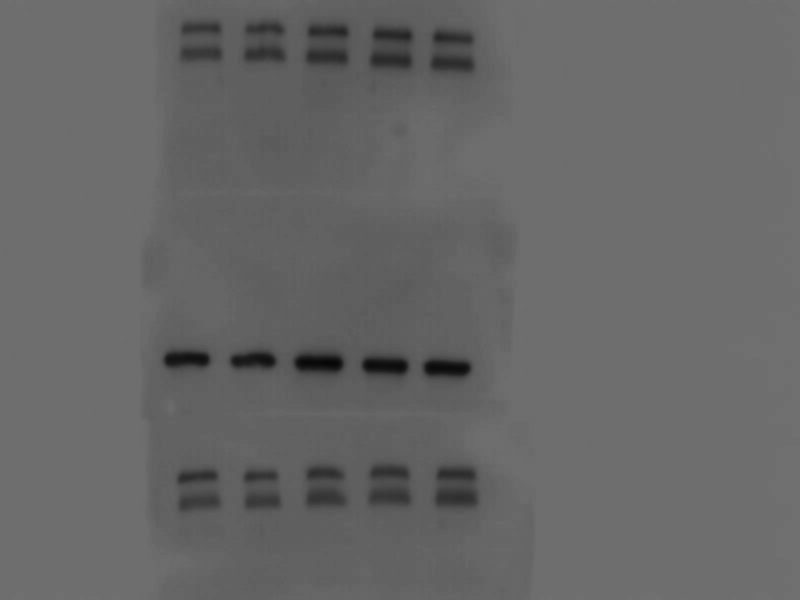

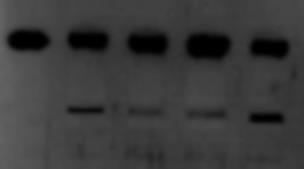

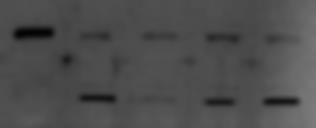


**Figure S8d. Apoptotic potential of CANE (0-125 μg/ml)- against PC-9 cells after 24 h of incubation.** d) Effect of CANE on expression of JNK, Bax, Bcl2, Cyt C, caspase-9, and 3 in PC-9 cells.

**CANE (125 μg/ml ) - - + +**

**Mitotempo - + + -**


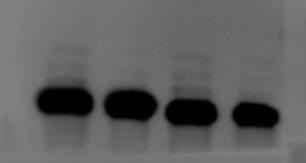


**Bcl2**


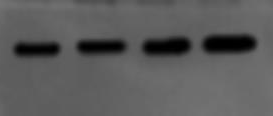


**Bax**


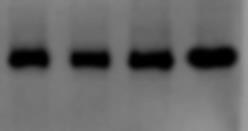


**Cyt C**

**Pr- Caspase 3**

**Cl. Caspase 3**


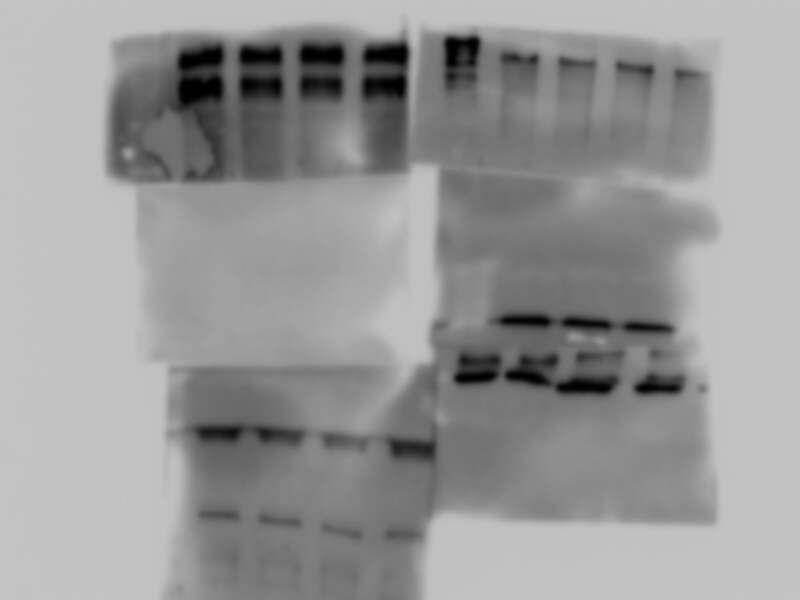


**JNK**

**p-JNK**

**β-actin**


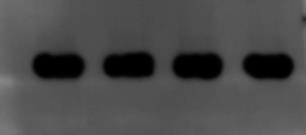


**Pr- Caspase 9**

**Cl. Caspase 9**


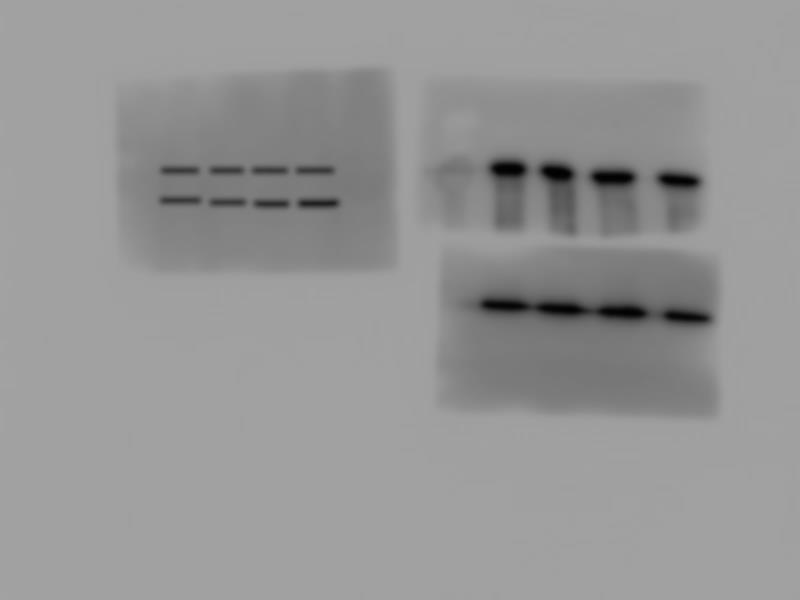

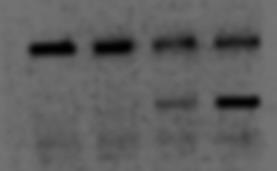

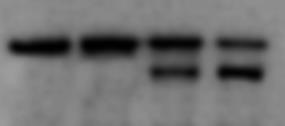


**Figure S8e. Apoptotic potential of CANE against PC-9 cells.** Cellular levels of protein markers JNK, p-JNK, Bax, Bcl2, Cyt C, caspase-3, caspase-9, and β- actin in PC-9 cells incubated with Mito-TEMPO (10 μM).

**Control 50 100**

**CANE (mg/kg)**


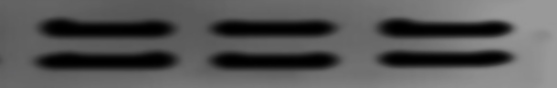

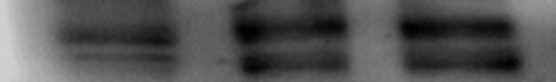

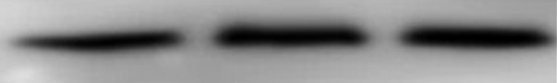

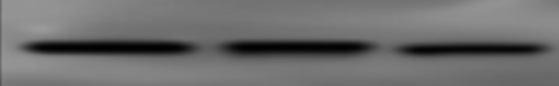

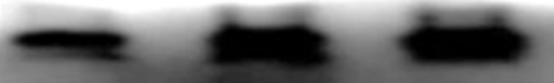

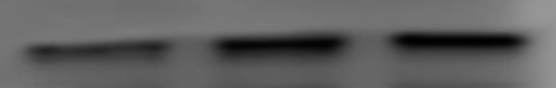

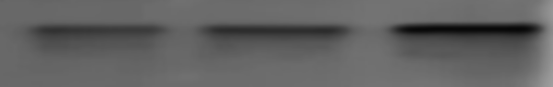

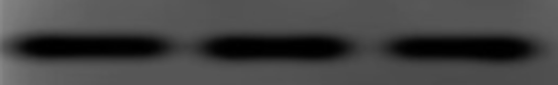


**Bax**

**JNK**

**β-actin**

**p-JNK**

**Bcl2**

**Cyt C**

**Cl. Caspase 3**

**Cl. Caspase 9**

**Figure S9f. CANE inhibits tumor growth.** Expression of JNK, p-JNK, Bax, Bcl2, Cyt C, caspase-3, caspase-9, and β- actin in tumor tissue.
